# The Role of Oat Nutrients in the Immune System: A Narrative Review

**DOI:** 10.3390/nu13041048

**Published:** 2021-03-24

**Authors:** Oliver Chen, Eunice Mah, ElHadji Dioum, Ankita Marwaha, Shobana Shanmugam, Nagappa Malleshi, Vasudevan Sudha, Rajagopal Gayathri, Ranjit Unnikrishnan, Ranjit Mohan Anjana, Kamala Krishnaswamy, Viswanathan Mohan, YiFang Chu

**Affiliations:** 1 Biofortis Research, Mérieux NutriSciences, Addison, IL 60101, USA; Eunice.Mah@mxns.com; 2 Friedman School of Nutrition Science and Policy, Tufts University, Boston, MA 02111, USA; 3 Quaker Oats Center of Excellence, PepsiCo Health & Nutrition Sciences, Barrington, IL 60010, USA; lHadji.Dioum@pepsico.com (E.D.); YiFang.Chu@pepsico.com (Y.C.); 4 PepsiCo Health & Nutrition Sciences, AMESA, Gurgaon 122101, India; ankita.marwaha@pepsico.com; 5 Madras Diabetes Research Foundation, Chennai, Tamil Nadu 600086, India; shobanashanmugam@mdrf.in (S.S.); malleshi@yahoo.com (N.M.); s2r_7@mdrf.in (V.S.); gayathri@mdrf.in (R.G.); drranjit@drmohans.com (R.U.); dranjana@drmohans.com (R.M.A.); kamala.krishnaswamy@gmail.com (K.K.); drmohans@diabetes.ind.in (V.M.)

**Keywords:** fiber, polyphenols, minerals, protein, human

## Abstract

Optimal nutrition is the foundation for the development and maintenance of a healthy immune system. An optimal supply of nutrients is required for biosynthesis of immune factors and immune cell proliferation. Nutrient deficiency/inadequacy and hidden hunger, which manifests as depleted nutrients reserves, increase the risk of infectious diseases and aggravate disease severity. Therefore, an adequate and balanced diet containing an abundant diversity of foods, nutrients, and non-nutrient chemicals is paramount for an optimal immune defense against infectious diseases, including cold/flu and non-communicable diseases. Some nutrients and foods play a larger role than others in the support of the immune system. Oats are a nutritious whole grain and contain several immunomodulating nutrients. In this narrative review, we discuss the contribution of oat nutrients, including dietary fiber (β-glucans), copper, iron, selenium, and zinc, polyphenolics (ferulic acid and avenanthramides), and proteins (glutamine) in optimizing the innate and adaptive immune system’s response to infections directly by modulating the innate and adaptive immunity and indirectly by eliciting changes in the gut microbiota and related metabolites.

## 1. Introduction

Optimal nutrition provides building blocks for the development and maintenance of all cells, including a well-functioning immune system. Huge global burden of morbidity and mortality is ascribed to malnutrition and includes both under and overnutrition. Malnourishment of a child or an adult makes them vulnerable to repeated infections and chronic inflammation, suggesting a relationship between malnourishment and immune defect [1]. In fact, the most common cause of immunodeficiency in the world is undernutrition [2], which manifests as depleted reserves or circulating concentrations of nutrients, reflecting chronic dietary inadequacies. While this condition is more prevalent in the underdeveloped and developing countries, it continues to be a problem in industrialized countries, especially among hospitalized/institutionalized individuals and the elderly, as well as people living in underprivileged communities where access to healthy foods is limited [3]. Moreover, detrimental to optimal immune function is hidden hunger, defined as a condition in which micronutrient intakes are inadequate but energy consumption is either adequate or excessive [4]. Chronic surplus intake of energy could modify immune response by disturbing the metabolic and endocrine status as well [5]. Nutrient inadequacies and deficiencies undermine the immune system, leading to immunosuppression and dysregulation of immune responses, such as impaired phagocytosis and decreased cytokine production [6,7]. Infection and illness can, in turn, increase nutrient losses through greater nutrient utilization, diminished appetite, and reduced nutrient absorption, resulting in a vicious cycle of undernutrition/hidden hunger and infection [2]. Thus, an adequate and balanced diet containing abundant varieties of foods, nutrients, and non-nutrient compounds is necessary to prevent infectious diseases, protect against infection-related complications, and support an effective immune response [7].

A dietary intake rich in plant-based foods with high quality and quantity of nutrients and non-nutrients such as vitamins, minerals, fiber, and polyphenolics may have a positive effect on the immune system. In this review, we principally discuss the potential contributions of oat as an integral part of a balanced diet and particularly that of oat nutrients (protein, copper, iron, selenium, and zinc) and bioactives (fiber and polyphenolics) to an optimal immune system and response. We explore the impact of selected nutrients in oats on immune health both directly by modulating the innate and adaptive immune system and indirectly by eliciting changes in the gut microbiota and related metabolites. The information reviewed here is significant particularly during the global SARS-CoV-2 (COVID-19) pandemic as people are more likely to consume energy dense food high in fats, refined carbohydrates, and sodium [8,9], which collectively impair the immune system and host defense against infections [10].

## 2. Immune System and Response

**Figure 1:**
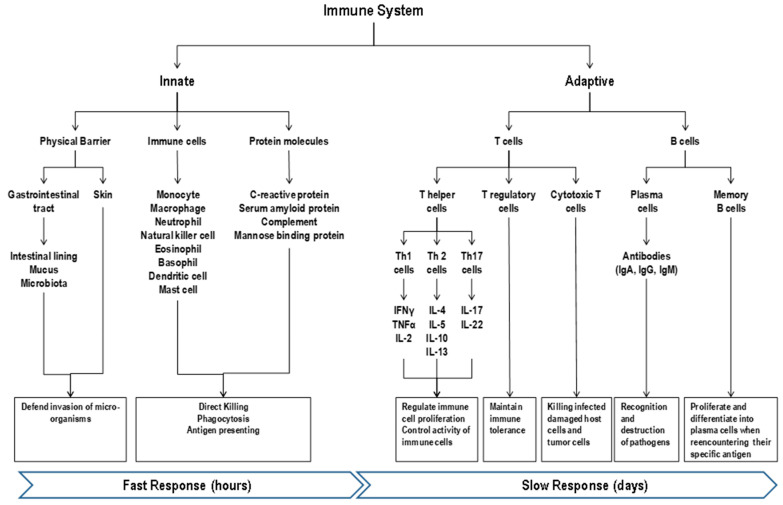
Overview of the immune response. The human immune system is comprised of both the innate and adaptive defenses. The innate immunity acts rapidly as the first line of protection to abate the establishment of overt infection with a goal for rapid elimination of infectious agents. The recognition of the presence of pathogens is mediated via the expression of nonspecific pattern-recognition receptors and not influenced by prior exposure and annihilation of invading pathogens occurs through direct destruction (complement system) and phagocytosis (immune cells). Although slow at first when encountering a microorganism for the first time, the adaptive immunity response is faster and stronger than the initial response when the microorganism is encountered again (i.e., re-infection) as it draws from its immunological memory from prior exposure to the antigenic components of the microorganism. Oats contain several nutrients that participate in both the innate and adaptive immune systems including fiber, micronutrients (e.g., zinc, iron, copper, and selenium), polyphenols, and proteins.

The human immune system is comprised of both the innate (fast, non-antigen specific) and adaptive (slower, antigen-specific) defenses [11] that work in an integrated, cooperative manner (Figure 1). The innate immune system includes epithelial barriers (skin and gut), cellular components [monocytes (macrophages), polymorphonuclear leukocytes (eosinophils and basophils), natural killer (NK) cells, neutrophils, mast cells, and dendritic cells (DCs)], and non-cellular soluble factors with antigen recognition molecules (C-reactive protein, serum amyloid protein, mannose-binding protein, antimicrobial peptides, complement, lysozyme, interferon, and other humoral factors) [12]. The innate immunity acts rapidly as the first line of protection to abate the establishment of overt infection typically via inflammatory processes with a goal for rapid elimination of infectious agents [7,13]. The recognition of the presence of pathogens is mediated via the expression of nonspecific pattern-recognition receptors and not influenced by prior exposure [13]. The innate immunity annihilates invading pathogens through two main mechanisms, direct destruction and phagocytosis [12,14]. The direct destruction is mediated by the complement system, attack of reactive radicals released by phagocytes, and toxic proteins released by NK cells. The complement system, comprising more than 30 proteins, elicits immune responses that eliminate invading pathogens by direct lysis or promoting phagocytosis [11]. Toxic proteins secreted by NK cells including perforin, proteases, and defensins can directly kill pathogens [15]. Phagocytosis of the innate immunity mainly exerted by monocytes, macrophages, neutrophils, eosinophils, and dendritic cells, is a cellular process for ingesting and degrading particles, including microorganisms, foreign substances, and apoptotic cells, via phagolysosome [11,16].

The adaptive system includes antigen-specific cells, namely B and T lymphocytes, with the former secreting antibodies specific to the infecting agents for humoral immunity and the latter with T helper [(Th) bearing CD4 receptor] cells coordinating the overall adaptive response, with cytotoxic T [(Tc) bearing CD8 receptor] cells killing virally-infected damaged host cells and tumor cells, and with T regulatory cells (Treg) being vital in maintaining immune tolerance to allow the immune system to ignore non-harmful non-self-substances (such as food, pollen, and environmental antigens) [6,13]. Intracellular fragments generated from phagocytosis of pathogens stimulate Tc cells and extracellular pathogens stimulate Th cells. The Th cells can be further categorized into three subtypes, defined by the cytokines they produce. Th1 cells produce interferon-gamma (IFN-γ), tumor necrosis factor-α (TNF-α), and interleukin (IL)-2 and play a crucial role in antiviral and cellular immune responses, Th2 cells produce IL-4, -5, -10, and -13 and are involved in humoral (antibody), anti-parasitic, allergic responses [17] and Th17 cells secrete IL-17A, IL-17F, and IL-22 and contribute to fighting extracellular pathogens [6,18]. After binding, cytokines can regulate growth, development or activity of the target cells. For example, TNF-α, IL-1 and IL-6 regulate monocytes and macrophages, stimulate acute phase protein synthesis in the liver, and suppress appetite [14]. B lymphocytes differentiate into plasma cells, producing a variety of antibodies, immunoglobulins (Igs), which facilitate recognition and destruction of pathogens [19]. For example, IgA present in the mucosal surface protects against bacterial and viral infections. Although slow at first when encountering a microorganism for the first time, the adaptive immunity response is faster and stronger than the initial response when the microorganism is encountered again (i.e., there is re-infection) as it draws from its immunological memory from prior exposure to the antigenic components of the microorganism. When an antigen binds to the small number of activated lymphocytes that recognize it, it can stimulate lymphocytes clonal division, increasing the immune response to the antigen, a process termed lymphocyte expansion or proliferation. This memory mechanism also supports the effectiveness of vaccination against subsequent pathogen exposure [13].

Assessing functionality and status of the immune system is complex and challenging because there is no single marker that reflect the health of the overall immune system, including innate and adaptive immunity [12]. In general, immune cell functions and populations and antibody and cytokine levels are measured to provide a view on individual elements of immunity but their relevance to clinical endpoints remains to be established. Additionally, the ambiguity of inflammatory status derived from acute infection-related inflammation vs. low-grade chronic inflammation commonly present in chronic diseases [20] can influence the accuracy of the assessment on clinical relevance. Thus, clinical endpoints such as infection rate and duration, symptom severity, and antibody response to vaccination are more widely accepted as evidence of an immune benefit of interventions than the changes in biomarkers. Nevertheless, the use of combinations of immune markers such as T-cell proliferation and production of Th1, Th2, and regulatory-type cytokines (e.g., IFN-γ, IL-2, IL-4, IL-5, IL-10, and TNF-α) has been commonly employed to evaluate immunity in research settings [12,21].

The immune system is particularly vulnerable to free radical attacks because the immune cells rely on cell-cell communications via receptors on membranes whose lipid-rich structures are susceptible to free radicals induced oxidation and consequent damages. Unfortunately, overwhelming amounts of free radicals are synthesized via respiratory burst during phagocytosis of pathogens [22]. Additionally, chronic inflammation is also linked to increased free radical productions resulting in higher oxidative stress. Thus, the adequate antioxidant defense system is absolutely needed to protect immune cells and other tissues from free radical attacks and related complications and damages.

## 3. Nutrition and Immune System and Functions

The immune system is influenced by genetic, physiological, and lifestyle factors, including age, sex, stress, hormone, exercise, drinking, smoking, health condition, and nutrition status [12]. Of these factors, nutrition is a modifiable factor that is fundamental to the development and maintenance of a healthy immune system [6,7]. The significance of nutrition in the robust, optimal immune system is manifested especially during malnutrition, including undernutrition and overnutrition. For example, protein deficiency increases the risk of infection due to low counts of antibodies and immune cells because amino acids are building blocks for the proliferation of immune cells and synthesis of immune effector molecules, [23]. Obesity, generally caused by overnutrition is linked with alterations in leukocyte development, phenotypes, and activity and the coordination of innate and adaptive immune responses [24]. Additionally, the impact of hidden hunger on the optimal immune response can be manifest even in healthy individuals.

The magnitude of the influence of nutrients on different components of the immune system does not always follow a linear dose-response relationship, with some being relatively insensitive to nutrient status or supply [14]. Moreover, the health condition and nutrient status of individuals can affect the response of the immune system to nutrient intakes. For example, healthy individuals with optimal nutrition may not benefit from supplementation of certain nutrients unless those statuses are deficient [14,25]. On the other hand, the increased intake of nutrients that are supportive of the immune system can confer immune benefits in people at increased risk of nutrient inadequacies and utilization, such as hospitalized and institutionalized patients, who are generally older. Along with increasing age, a variety of structural and functional changes in the immune system occur, such as increased levels of circulating pro-inflammatory cytokines, and insufficient production of naive immune cells. Furthermore, the amplified oligo-clonal expansion of memory immune cells, leading to less effective innate and adaptive immune responses, increased risk of auto-immune response, and increased susceptibility to infections (immunosenescence) [12]. All these changes support the importance of optimal nutrition for functional immunity for fighting infectious diseases.

Nutrients that play a larger role in the immune system can be regarded as immunomodulators, which can be further categorized into immunoadjuvants, immunostimulants, and immunosuppressants [26]. Immunoadjuvants are specific immune stimulators enhancing the efficacy of vaccines. Immunostimulants activate the mediators or components of the immune system, whereby they can enhance resistance against infection, autoimmunity, cancer, and allergy. Immunosuppressants can inhibit the immune system, whereby they can be used to control the pathological immune reaction subsequent to organ transplantation. Micronutrients, including vitamins A, C, D, B6, B9, and B12, copper, iron, selenium, and zinc, collectively function to support the development and maintenance of the immune system. These micronutrients can be considered an immunostimulant. However, the prevalence of deficiency or inadequacy of some of these nutrients is common, such as zinc [27,28,29], iron [30], and selenium [28,29]. A variety of mechanisms enabled by these nutrients that can contribute to modulation of the immune system include (1) production and activity of antimicrobial proteins; (2) growth, differentiation, and motility/chemotaxis (homing) of immune cells; (3) phagocytic and killing (e.g., oxidative burst) activities of neutrophils and macrophages; (4) promotion of and recovery from inflammation (e.g., antioxidant activity); and (5) cytokine and antibody production [6,7,14,31]. As symptomatic infections can be a result of compromised immune functions consequential to deficient micronutrient stores [25,32], maintaining optimal nutrient status is paramount for humans defending against infectious diseases, including cold/flu and COVID-19.

## 4. Nutrient Composition of Oats

A variety of plant-based foods provide functional benefits due to their contents of vitamins, minerals, polyphenolics, terpenoids, alkaloids, sterols, pigments or unsaturated fatty acids [32]. Therefore, these foods may help optimize immunity that can mount an effective reaction against infections and/or manage inflammatory damages in a controllable manner, both crucial for the resolution of infectious diseases. Oats contain numerous nutrients and bioactives that have been associated with anti-inflammatory anti-oxidant and immunogenic responses [33].

Oats (family of *Poaceae* or *Graminae*), classified under the genus *Avena*, have 27 known species or sub-species. *Avena sativa*, also referred to as cultivated oat, is the most economically important species worldwide. Oats are a nutritious whole grain and mainly supply carbohydrate in the form of starch, have reasonably high lipid levels, and contain several of micronutrients, vitamin B1, B6, folate, pantothenic acid, Mn, Mg, Se, Fe, Zn, and Cu [31] (Table 1). Additionally, oats contain a higher protein content compared to widely consumed cereals, such as corn and rice, with a fairly good balance of essential amino acids [34]. After dehulling, the fiber content of oat groats is decreased to 10–12%, with roughly 40% as soluble fiber, mainly β-glucan, and 60% as insoluble fiber, composed primarily of cellulose, hemicellulose and lignin [35]. β-glucans in oats consist of linear branched linkage of 30% β-1,3 glucan and 70% β-1,4 linked β-D-glucopyranosyl units [35]. Among β-glucan containing foods, oats are by far the predominant dietary source [34]. The amount of β-glucan in oats ranges from 2 to 8 g/100 g [36] and the total and soluble β-glucan contents of oats was reported to be on average 4.40 and 3.88 g/100 g, respectively [37]. Nutrient contents in oats, like all other plant foods are variable and influenced by a long list of factors, including cultivar, location, cultivation, season, and postharvest processing [35].

Oats have been used traditionally as a stimulant, antispasmodics, antitumor, diuretics or neurotonics [38]. Oat constituents display antioxidant, anti-inflammatory, wound healing, immunomodulatory, antidiabetic, and anticholesterolemic activities that are protective against acute and chronic illnesses [38]. According to the FDA and EFSA nutrient source guidance, oats can be a good source of protein, fiber, iron, magnesium, phosphorus, zinc, copper, manganese, and selenium (Table 2). Oats per 40-g serving is a “good” or “excellent” source of nutrients for protein, fiber, iron, magnesium, phosphorus, zinc, copper, manganese, and selenium, based on the US FDA nutrition claim regulation or “a source of nutrients” based on the EFSA regulation. According to the US FDA describe the level of a nutrient in foods, a food containing at least 10–19 and 20% more of the Reference Daily Intake (RDI) for micronutrients or of the Daily Reference Value (DRV) for protein, dietary fiber, or potassium per reference amount customarily consumed can be labeled with “good source of” and “excellent source of”, “high” or “rich in”, respectively [39]. In the European Union (EU), a nutrient source claim can be used to label a food high in fiber when the product contains at least 6 g of fiber per 100 g or at least 3 g of fiber per 100 kcal [40]. Furthermore, a food is a source of protein when at least 12% of the energy value of the food is provided by protein, and a claim that a food is a source of vitamins or minerals can be made where the product contains 15% of the recommended allowance as defined in the Annex to Directive 90/496/EEC [40].

In addition to containing essential nutrients and dietary fibers, oats contain a list of polyphenolics, particularly phenolic acids and avenanthramides. Among 22 commercial oat products (oat bran concentrate, oat bran, flaked oats, rolled oats and oatcakes) analyzed using HPLC-DAD assay, the total concentrations of phenolic acids and avenanthramides ranged from 0.4 to 1.5 mg/g, with oat bran concentrate having the highest [41]. This translates to 16 to 25 mg total phenolic acids and 1–2 mg avenanthramides in a 40 g serving of commercial oat products. Like other cereals, maize, whole-grain wheat, and rice, ferulic acid is the predominant polyphenolics in oats with the content ranging from 9 to 15 mg in a 40 g serving [41].

A poor diet with inadequate intake of essential micronutrients and fibers plays a major role in immune dysfunction, leading to a state of systemic chronic inflammation (SCI). This state is well accepted as a major contributor in the development of non-communicable chronic diseases. The typical Asian Indian diet is reported to be low in protein (9–10% of total daily caloric intake) and high in carbohydrates (70–80% of total daily caloric intake), mainly in the form of refined carbohydrates, such as white rice and white flour, putting these populations in a growing risk of non-communicable diseases such as type 2 diabetes. Globally, the high prevalence of type 2 diabetes and prediabetes, especially in China, India and other emerging countries is exacerbated by a low diet quality and a higher prevalence of obesity that coexists with that of type 2 diabetes. Observational studies have shown that whole grains intake is associated with weight loss, reduced insulin resistance and type 2 diabetes [42,43]. Substituting refined grains with wholegrains like oats improves carbohydrate quality with higher fiber, protein and micronutrients like Zn, Se, Fe and polyphenols can improve the overall diet quality and potentially promote a functional immune system. In the following sections, we discuss potential benefits of oat nutrients that are a good source (identified based on the US and EU nutrient claim) to the immune system with special emphasis on immunity against infection. Additionally, the contribution of oat polyphenolics to the immune system is discussed as the benefits of these phytochemicals on the immunity have been well appreciated.

## 5. β-Glucans and Other Dietary Fibers

Dietary fibers are mainly polysaccharides that cannot be digested and absorbed in the human gastrointestinal tract [44]. Historically, dietary fibers are known for reducing energy intake and helping body weight loss and maintenance, as well as for modulation of the gut microbiota [45]. Recently, their effects on the immunity and the incidence/severity of infectious diseases are recognized, and this relationship is probably mediated by the gut microbiota [46]. The underlying mechanism of actions of dietary fibers on the immune system and risk of infectious diseases have not been elucidated, although four probable mechanisms have been proposed [45,47]. First, the fermentable fibers can spare mucin from being utilized by the gut microbes during fermentation. Mucin is part of the innate immunity serving as the physical barrier against the invasion of infectious microorganisms. Second, dietary fibers contribute to the maintenance of the gut microbiota with diverse composition and variable, complement metabolic functions that can abate the proliferation of opportunistic pathogens, which cause inflammation and weaken the immune system. Third, short-chain fatty acids (SCFAs), microbially derived metabolites of dietary fibers, stimulate mucus and anti-microbial peptide productions, increase expression of tight junction proteins, and modulate innate and adaptive immunity. Fourth, dietary fibers can benefit the overall inflammatory status indirectly by blunting the glycemic load and postprandial glucose-induced systemic inflammation observed in pathological states like type 2 diabetes [47].

There is growing evidence illustrating the effect of SCFA on the immunity. Human and animal studies show that butyrate inhibits proinflammatory cytokines IFN-γ, TNF-α, IL-1β, IL-6, IL-8, and IL-12 and up-regulates IL-10 and TGF-β partly through the inhibition of NF-κB signaling pathway [7,48]. Additionally, SCFAs promote the differentiation of naive T cells into Th1 (playing a crucial role in antiviral and cellular immune responses) and Th17 cells [45]. Moreover, SCFAs are capable of inhibiting the expression of toxic genes of pathogens, such as *Salmonella, E. coli,* and *Campylobacter jejuni* [49]. Although these putative actions of SCFAs are more relevant to the intestinal immunity, their effects are anticipated to extend to other parts of the human body, such as the respiratory system. In line with this notion, dietary fiber consumption is inversely linked to the risk of mortality from respiratory and infectious diseases [50]. It is possible that the fortified intestinal integrity by SCFA can ward off the invasion of pathogens or their residues, such as lipopolysaccharides, and absorbed microbial metabolites, which after entering the body, can affect systemic immune responses [51]. Additionally, the linkage of the gut microbiota and respiratory infection is substantiated by the positive results of human trials testing the effect of *Lactobacillus* spp. and *Bifidobacterium* spp.-based probiotics on respiratory infections in humans and the efficacy of influenza vaccination [52,53,54]. As dietary fibers can increase the abundance of normally recognized beneficial bacteria including *Lactobaccillus* spp., *Bifidobacterium* spp., *Prevotella* spp., *Ruminococcus* spp., *Facealibacterium* spp., in the human gut [55,56,57], their favorable effects on the immune system and the risk of infectious diseases via the modification of the gut microbiota composition is expected [7,58]. Additionally, the favorable changes in the gut microbiota may help fight viral infection by blocking cell internalization, destabilizing virion structure, and suppressing viral replication [7].

Dietary fibers can be grouped into different categories, such as prebiotic vs. non-prebiotic, soluble vs. non-soluble, fermentable vs. non-fermentable, and natural vs. synthetic. With a wide diversity of chemical structures, the biological effects of each dietary fiber are anticipated to be different. Beta-glucans are non-digestible polysaccharides naturally present in foods, such as oats, barley, bacteria, yeast, algae, and mushrooms [59]. Among cereals, the barley and oats have the highest β-glucan content. The biological activities of β-glucans are structure-dependent. The immune-modulating activities of β-glucans are present in those with (1,3)-β-linked backbone containing small numbers of (1,6)-β-linked side chains [60]. Even though oat β-glucans have either (1,3) or (1,4)-β-linkage, they display a modest immune effect. A cell culture study showed that the mRNA expression and production of TNF-α and IL-6 were significantly increased in THP-1 cells primed first with oat β-glucans before the challenge with LPS, suggesting oat β-glucans could enhance the responsiveness of the innate immune system [61]. However, in a human trial with trained male cyclists, supplementing oat β-glucans (5.6 g/d) for 18 days did not affect chronic resting or exercise-induced changes in immune function (NK activity, polymorphonuclear respiratory burst activity, lymphocyte proliferation, IL-6, IL-10, IL-1 receptor agonist, and IL-8) or URTI incidence during the 2-wk period after an intensified exercise [62]. In contrast, the results of two mouse studies conducted by the same research group showed that oat β-glucans decreased the susceptibility to respiratory infection following one bout of exercise stress [63,64]. Moreover, oral treatment of oat β-glucan extract protected mice against infection of pathogens, *Staphylococcus aureus* and *E. vermiformis* [65]. The effect of oat β-glucan on the gut microbiota is supported by the observed increase in *Bacteroides* and decrease in *Enterobacteriaceae* families in an in vitro human fecal fermentation experiment [66]. However, in a human study with elderly subjects with low habitual fiber intake, neither oat β-glucans nor arabinoxylans (12 g/day for 6 weeks) affected intestinal permeability and the gut microbiome, compared to placebo [67]. Moreover, oat β-glucan did not affect inflammatory markers in patients with hypercholesterolemia [68,69](Theuwissen et al., 2009; Queenan et al., 2007). Thus, more human studies are needed to demonstrate whether oat *β*-glucans can modulate immunity and inflammatory status via the gut microbiota.

Oats also contain insoluble fibers, particularly cellulous, lignin, and hemicellulose. Dietary cellulous is not fermentable in the colon and its potential immune benefits may be attributed to the resulting changes in the composition of the gut microbiota [70](Holscher 2017), which are involved in the local and systemic immunity and inflammation [46]. While clinical evidence on the impact of these fibers on the immunity is lacking, scarce preclinical data support their potential contributions. An endotoxemic mouse study showed that a high cellulose diet (30% by weight) decreased the number and activation of splenic macrophages and dendritic cells and amplified the suppressive function of T-regulatory cells after lipopolysaccharide administration [71] (Di Caro et al., 2019). The diet also led to an increased abundance of *Lachnospiraceae* and *Akkermansia.* The effect of oat insoluble fibers in oats on inflammation has been reported in a few human studies. The OptiFiT trial with 180 adults with impaired glucose tolerance showed that supplementation 15 g/d purified oat insoluble fibers containing 70 wt% cellulose, 25 wt% hemicellulose and 3–5 wt% lignin for two years did not affect CRP and IL-18 as compared to placebo even though it improved glycemic metabolism in people with impaired fasting glucose [72,73] (Honsek et al., 2018; Kabisch et al., 2019). More human studies are needed to demonstrate the effect of oat insoluble fibers on immunity and inflammation.

Oats are a good source of dietary fiber as one serving (40 g) provides 3.97 g dietary fiber. In developed countries, fiber intake is generally low, around 15–20 g/d [74], which is inadequate for maximizing the benefits of dietary fibers on the immune system. For example, a significant reduction in C-reactive protein (CRP), an acute phase protein and a marker of systematic inflammation, is noted with an increase in total fiber intake to 30 g/d [75]. Increased whole-grain intake (even below 5 g/d) has been associated with decreased CRP, IL-6, and TNF-α and increased SCFA [7]. Additionally, increased dietary fiber intake is associated with reduced mortality rates in respiratory-related diseases and improved lung function [46]. All these studies support the incorporation of fiber-rich oats to a healthy diet for immune health.

## 6. Minerals

Oats are good sources of several minerals known to play important roles in the immune system and these include the metal ions iron, zinc, and copper, which are recognized as contributing to a normal function of the immune system by EFSA [76,77,78]. Zinc and iron deficiencies are associated with deleterious health outcomes, such as increased susceptibility to infections and consequent morbidity and mortality [12,75,76]. Unfortunately, the global prevalence of zinc deficiency has been estimated to be 30% [79] while iron deficiency ranks number nine among 26 risk factors included in the Global Burden of Disease 2000 project, and accounts for 841,000 deaths and 35,057,000 disability-adjusted life years lost [80]. In contrast, copper deficiency is very rare and is often associated with genetic disorders that interfere with copper absorption, or special circumstances such as poor enteral nutrition [81]. While there are many interlinking factors contributing to iron deficiency, one factor is the low bioavailability of iron and zinc from cereals, which comprises 40–60% of total daily energy intakes in developed countries and up to 80% in certain developing countries [82]. Despite being good sources of iron and zinc, cereals have high phytic acid content (Table 3) that hinders their bioavailability. Studies using cereal porridges made from rolled cereals estimated iron absorption between 0.33% for oats and 1.8% for maize [83]. Removal of phytic acid resulted in a 3–12 times increase in iron absorption; specifically, absorption of iron from oats increased over 8-fold [83]. For the most part, commercially available oat products are not dephytinized, although some, such as oat groats, have lower phytic acid content due to the removal of the hull [84]. Additionally, many cereal products are fortified with iron. Other strategies that consumers may employ to decrease phytic acid content and thus increase iron and zinc availability include soaking oats overnight prior to consumption [85]. Unlike iron and zinc, copper absorption is not affected by phytic acid.

Selenium also support the immune system indirectly via its role in the antioxidant defense system [12]. It is very well appreciated that an increase in free radical level resulting from selenium deficiency increased the risk of viral and bacterial infections. This is partially due to the increased mutation of viruses such as Coxsackie and influenza that can be more virulent [87]. In a recent small observational study with 50 hospitalized patients with COVID-19, selenium deficiency was found in 42% of patients, suggesting selenium status may affect the onset and severity of COVID-19 [88]. Moreover, selenium deficiency is associated with over-expression of influenza-induced proinflammatory cytokines [89,90]. Unfortunately, concentrations of selenium from plasma, serum or whole blood of healthy adults from 69 countries suggest nutritional selenium deficiency is prevalent in more than half of the countries listed [91]. Cereals and cereal products, especially those grown in selenium-rich soils, is a major contributor of dietary selenium globally [92]. All plants accumulate selenium, and this includes cereal crops. The amount of selenium in different types of cereals is directly related to selenium bioavailability in the soil and dependent on plant genetics [93]. Selenium from cereals, including oats, is highly bioavailable [94,95].

## 7. Polyphenolics

Whole grains including oats are a rich source of phytochemicals that confer health benefits. While there is scarce evidence of whole grain consumption on the immunity or infection, the data of two human trials show that compared to refined grain products, whole grain consumption increased the percentage of terminal effector memory T cells [96,97]. These results suggest nutrients and non-nutrient phytochemicals in whole grain augment an adaptive immune response to a recall antigen. During the processing to produce refined grains, most phytochemicals in whole grains are lost substantially but this does not happen to oats because oats are typically consumed as a whole grain. Among thousands of phytochemicals, polyphenolics, including phenolic acids, flavonoids, and others, have been well appreciated for their antioxidant, anti-inflammatory, anti-allergic, and immunomodulating activities [98]. These activities are partially mediated through their regulatory effects on antioxidant defense system and innate and adaptive immunity via inhibition of NF-κB and AP-1 and activation of Nrf2 [99,100]. Additionally, actions enabling a reduction in viral infections include suppressing neuraminidase and hemagglutinin activity, decreasing viral replication, hemagglutination, adhesion and penetration, and modifying cellular signaling pathways and transcription factors [7,101]. These antiviral actions were illustrated in an in vitro study showing that a number of flavonoids diminished infectivity and replication of herpes simplex virus type 1 (HSV-I), polio-virus type 1, parainfluenza virus type 3 (Pf-3), and respiratory syncytial virus (RSV) [102]. Clinical evidence in this regard remains scarce. A human trial conducted in healthcare professionals with an increased risk for cold/flu via patient contacts showed that a fruit/vegetable extract supplement rich in polyphenolics decreased by 20% of moderate or severe common cold symptom days [103]. This benefit is supported by a rodent experiment, in which a polyphenol extract from *Cistus Incanus* lowered the influenza virus infection rate and reduced mortality of mice [104]. While polyphenolic compositions are variable between foods, it can be anticipated that polyphenolics in oats may have a similar effect on the immune system, but more clinical evidence is warranted [99].

Oats contain polyphenolics bioactives, particularly phenolic acids and avenanthramides. These non-nutrient phytochemicals contribute to health benefits of oat consumption, including immune health. The preponderant polyphenol in oats, ferulic acid, has been shown to associate inversely with 35% lower risk with elevated CRP status (>3 mg/L) in a cross-sectional study with 315 participants of the EPIC [105]. This result implicates a potential effect of consumption of ferulic acid-containing foods on the immune system. While clinical evidence of ferulic acid on the immune system remains scarce, promising data have been observed in rodent studies. For example, ferulic acid improved survival rate and mitigated weight loss in mice infected with influenza virus A/FM/1/47(H1N1) by activating toll-like receptor (TLR)-7 and -9, increasing production of type I IFNs, and inhibiting NF-κB pathway [106]. In another rodent study, ferulic acid pretreatment alleviated pulmonary histological changes, function, and inflammation in rats with LPS-induced acute respiratory distress syndrome [107]. Thus, these data are in keeping with a previous review indicating that natural phenolic acids possess an inhibitory effect on infection of HIV, hepatitis B and C virus, herpes simplex virus, influenza virus, and respiratory syncytial virus [108]. While there is no human data demonstrating the effect of ferulic acid on acute infection, its inflammatory modulating action was noted in human trial showing that as compared to the control, ferulic acid supplementation (1 g/d for 6 weeks) significantly reduced inflammatory markers CRP and TNF-α in adults with hyperlipidemia [109]. Avenanthramides are polyphenolics unique to oats and display anti-inflammatory effects. Using an acute eccentric exercise-induced inflammation model, avenanthramide supplementation (20.6 mg/day for 8 weeks) reduced circulatory inflammatory cytokines and inhibited expression of chemokines and cell adhesion molecules in 24 young adults [110]. Similarly, avenanthramides (9.2 mg/d for 8 weeks) blunted acute eccentric exercise-induced neutrophil respiratory burst in 16 young women. Such an effect of avenanthramides on neutrophil respiratory burst was also noted in 16 older women [111]. These results indicate that avenanthramides are capable of mitigating exercise-related inflammation but their effect on the immunity against infection remains to be examined.

Potential mechanisms explaining the link between ferulic acid and avenanthramides and immune system include boosting antioxidant defense system, as well as modulating inflammation via increasing histone deacetylase activity, regulating transcription factors, and attenuating endoplasmic reticulum stress signaling [105]. Another mechanism is mediated through IFNs, which contribute to inhibiting viral replication including SARS coronavirus via the activation of TLR-7 [112]. Interestingly, ferulic acid was also reported to up-regulate the activation of TLR-7 and stimulation of the type 1 IFN via the induction of heme oxygenase-1 [113]. Additionally, TLR4 is involved in the alleviation of excessive inflammatory symptoms induced by a viral infection, and its expression is abated by ferulic acid [114].

In addition to direct interactions with the immune systems, polyphenolics can regulate the immunity through indirect mechanisms. Polyphenolics are not well absorbed in the small intestine and remain intact in the colon where they have dynamic interactions with the gut microbiota [115]. Since the emerging evidence shows the link between immunity and infection and the gut microbiota, it can be anticipated that polyphenolics with a microbial modulating activity can affect immunity [46,116,117,118]. Uncontrolled excessive oxidative stress during infection has been implicated in tissue injuries, such as lung tissue injury and epithelial barrier dysfunction in acute respiratory viral infections. Given their up-regulated effects on the antioxidant defense, polyphenolics are likely to help ameliorate free radical induced complications [7,46]. Finally, the results included in a US patent application shows that avenanthramide 2c is a promoter of iron bioavailability in humans [119], suggesting this polyphenol can regulate the immunity via its influence on the iron status (iron and immunity reviewed above). All these mechanisms implicate the potential of oat polyphenolics for sustaining optimal immunity against infectious diseases.

## 8. Proteins and Glutamine

Proteins are crucial for the production of immune cells and effector molecules such as antibodies and it has been estimated that up to 60 g protein is utilized daily during an episode of infection [120]. Not surprisingly, people with a low protein status are more susceptible to infection or have a lower antibody titer response after vaccination than those with an adequate protein status [7,121]. As such, maintaining optimal protein homeostasis is very important to sustain optimal immunity, and consuming an adequate amount of high-quality protein during infection is critical to restrain symptomatic severity and complications [120]. The protein sources affect immunity and infection to a different degree due to content of amino acids, especially those with immunomodulating properties. Among the amino acids, arginine, glutamine, and tryptophan are known for modulating the immune system [99]. Of these, glutamine is recognized as important for the “maintenance of the normal function of the immune system” by the European Food Safety Authority [122]. Glutamine regulates the proliferation of lymphocytes, neutrophils, and macrophages through (JNK) signal-regulated kinases and activator protein (AP)-1 signal transduction pathways and controls the production of various cytokines, such as IL-6, IFN-γ, and TNF-α [7,123]. Additionally, activated B and T cells have an increased demand for glutamine for the production of IFN-γ and IL-2 and T cell proliferation [99,124]. Recent data showed that glutamine supplementation and metabolism is a vital part of the immune function and greatly reduces the risk of infectious complications in critically ill or surgical patients [125,126]. Oats, including all oat products listed in Table 1, contain proteins at 12–13% dry weight, equivalents to at least 5.0 g protein per 40-g serving and provision of 8.9% of daily protein requirement of 0.8 g/kg body weight for a 70-kg American male adult (Table 1 and Table 2). However, the protein quality score as assessed using Digestible Indispensable Amino Score (DIAAS) in oats is 57 which is below the threshold (DIAAS < 75) of being able to make protein claims [127]. Nevertheless, A. sativa oat groats are abundant in glutamine/glutamic acid, 23.9% of total protein in oats, delivering 1.63 g glutamine per serving [128]. While the amounts of glutamine administered in clinical studies for immunity and diminishing infectious complications are much larger than the amount in one serving of oats, the incorporation of oats to a healthy diet pattern is anticipated to help maintain the pool of this amino acid for immunity support, as well as to support protein status for production of immune cells and antibodies.

## 9. Conclusions

The human immune system is comprised of both the innate and adaptive defenses that work in an integrated, cooperative manner. Nutrient inadequacies/deficiencies can undermine the immune system, thereby increasing the risk of infectious diseases, as well as non-communicable diseases such as diabetes, and aggravating consequent symptom severity and complications if infected. Thus, maintaining optimal nutrient status is paramount for humans defending against infectious diseases, including cold/flu and COVID-19. Micronutrients, including vitamins A, C, D, B6, B9, and B12, copper, iron, selenium, and zinc, as well as dietary fibers and non-nutrient phytochemicals, such as polyphenolics, collectively function to support the development and maintenance of the immune system and are being considered an immunostimulant. Oats contain several of these immunomodulating nutrient summarized in Table 4. In this narrative review, we discussed the contribution of copper, iron, selenium, and zinc, four elements that oats are either a good or excellent source, to the immune system against infectious diseases. Additionally, the abundance of protein, as well as glutamine, an amino acid known for reducing infectious complications, support the consumption of oats for maintaining the pool of glutamine for immunity support. Oats are a renowned source of dietary fibers and β-glucans. While these molecules may directly modulate the immune system, they can also improve/maintain immunity through indirect mechanisms, such as modifying the gut microbiota composition and functions and increasing the production of SCFAs. Finally, polyphenolics, including ferulic acid and avenanthramides in oats, can help optimize the immune system by regulating inflammatory response, boosting the antioxidant defense system, and modulating the gut microbiota. Therefore, oats are a good source of numerous nutrients, including fiber (β-glucans), copper, iron, selenium, zinc, glutamine, and polyphenolic bioactives (ferulic acid and avenanthramides) that can help optimize the immune system and response to infections, including cold/flu viruses and other pathogens. These nutrients support the immunity directly by modulating the innate and adaptive immune system and indirectly by eliciting changes in the gut microbiota and related metabolites.

## Figures and Tables

**Table 1:** Contents of macronutrients and micronutrients of in 100 g of raw Oats and Oat products in comparison with other common cereals as reported in the USDA Food Data Central database.

Nutrients (per 100 g)	Raw White Rice	Raw Sweet Yellow Corn	Raw Whole Wheat Flour	Regular Quick Oat ^1^	Old Fashioned Oat ^1^	Steel Cut Oat ^1^	Instant Oat ^1^	Whole Oat Flour ^2^	Oat Bran ^2^
USDA Food Data Central ID	169760	169998	790085	173904	980451	1015405	685984	368827	168872
Energy (kcal)	360	86	370	379	375	375	375	400	246
Protein (g)	6.61	3.27	15.1	13.15	12.5	12.5	12.5	17.5	17.3
Total Fat (g)	0.58	1.35	2.73	6.52	7.5	6.25	7.5	7.5	7.03
Carbohydrate (g)	79.34	18.7	71.2	67.7	67.5	67.5	67.5	65	66.22
Total Fiber (g)	N/A	2	10.6	10.1	10	10	10	10	15.4
Calcium, (mg)	9	2	38	52	50	N/A	45	50	58
Iron (mg)	0.8	0.52	3.86	4.25	3.75	4.5	3.5	4.5	5.41
Magnesium (mg)	35	37	136	138	100	N/A	N/A	N/A	235
Phosphorus (mg)	108	89	352	410	325	N/A	N/A	N/A	734
Potassium (mg)	86	270	376	362	N/A	N/A	N/A	N/A	566
Zinc (mg)	1.16	0.46	3.24	3.64	N/A	N/A	N/A	N/A	3.11
Copper (mg)	0.11	0.054	0.452	0.391	N/A	N/A	N/A	N/A	0.403
Manganese (mg)	1.1	0.163	3.56	3.63	N/A	N/A	N/A	N/A	5.63
Selenium (μg)	N/A	0.6	23.6	28.9	N/A	N/A	N/A	N/A	45.2
Thiamin (mg)	0.07	0.155	0.504	0.46	0	N/A	N/A	N/A	1.17
Vitamin B6 (mg)	N/A	N/A	N/A	0.1	N/A	N/A	N/A	N/A	N/A
Folate, DFE (μg)	9	42	39	32	N/A	N/A	N/A	N/A	52
Pantothenic acid (mg)	1.34	0.717	N/A	1.12	N/A	N/A	N/A	N/A	1.5
Choline (mg)	N/A	N/A	N/A	40.4	N/A	N/A	N/A	N/A	32.2
Glutamic acid (g)	1.288	0.636	N/A	2.83	N/A	N/A	N/A	N/A	3.748

Reference: USDA FoodData Central database, accessed on 8 October 2020 and 23 November 2020 (https://fdc.nal.usda.gov/fdc-app.html#/?query=oat). ^1^ Old fashioned oats are the oat groats steamed and rolled into flakes. Quick oats are oats that have been steamed longer than old fashioned oats and rolled extremely thin. Instant oats are similar to quick oats but oats are cut smaller and rolled thinner than quick oats. Steel cut oats are oat groats that are steel cut. ^2^ Oat flour are oat groats that are ground. Oat bran is the outer casing of the oat groat. Abbreviation: N/A, not available.

**Table 2:** Recommended dietary allowance of the nutrients that can be found in appreciable amounts in oats.

	WHO	EFSA	US	India	China	Mexico
Protein (g)	0.83 g/kg BW	≥12% of total kcal	M: 56 F: 46	M: 60 F: 55	M: 65 F: 55	1 g/kg BW
Total Fiber (g)	N/A	25	M: 28–34 F: 22–25	30	25–30	M: 30–35 F: 26–30
Iron (mg)	M: 9.1–27.4 F: 7.5–58.8	M: 11 F: 11–16	M: 8 F: 8–18	21	M: 12 F: 12 -20	17
Magnesium (mg)	M: 224–260 F: 190–220	M: 350 F: 300	M: 400–420 F: 310–320	M: 340 F: 310	310–330	248
Phosphorus (mg)	N/A	550	700	600	670–720	664
Potassium (mg)	≥ 3510	3500	4700	M: 3750 F: 3225	2000	N/A
Zinc (mg)	M: 4.2–14 F: 3–9.8	M: 9.4–16.3 F: 7.5–12.7	M: 11 F: 8	M: 12 F: 10	M: 12.5 F: 7.5	10
Copper (µg)	N/A	M: 1600 F: 1300	900	3000	800	650
Manganese (mg)	N/A	3	M: 2.3 F: 1.8	2–5	4.5	N/A
Selenium (µg)	M: 33–34 F: 25–26	70	55	40	60	41

Values are Recommended Dietary Allowance reflecting the average daily level of intake sufficient to meet the nutrient requirements of nearly all (97–98%) healthy people. Abbreviations: BW, body weight; EFSA, European Food Safety Authority; F, females; g, gram, kg, kilogram; M, males, mg, milligram; µg, microgram; WHO, World Health Organization.

**Table 3:** Phytic acid content of several major cereals ^1^.

Major Cereals	Phytic Acid g/100 g Dry Weight
Barley	0.38–1.16
Sorghum	0.57–3.35
Oat	0.42–1.16
Rye	0.54–1.46
Millet	0.18–1.67

^1^ The information is obtained from the cited study [86].

**Table 4:** Mechanism of actions by which oat constituents modulate the immune system against infection.

Constituents	Actions
Fiber	Spare mucin (physical barrier against infection) from being utilized by the gut microbes Create an ecosystem unfavorable for survival of pathogens in the gut Substrate for production of SCFAs which promote gut integrity, exert anti-inflammatory action, regulate differentiation of naive T cells into Th1 and Th17 cells B-glucans enhance the responsiveness of the innate immune system
Copper, iron, selenium, zinc	Cofactors of antioxidant enzymes Protect immune cells from free radical attacks Regulate proliferation of immune cells
Polyphenols (e.g., ferulic acid, avenanthramides)	Regulate antioxidant defense system and innate and adaptive immunity via inhibition of NF-κB and AP-1 and activation of Nrf2 Display anti-inflammatory via increasing histone deacetylase activity, regulating transcription factors, and attenuating endoplasmic reticulum stress signaling Exert antiviral action suppressing neuraminidase and hemagglutinin activity, decreasing viral replication, hemagglutination, adhesion and penetration Increase IFNs, which contribute to inhibiting viral replication including SARS coronavirus via the activation of TLR-7 Promotes iron bioavailability in humans
Proteins	Substrate for proliferation and immune cells and molecules Regulates the proliferation of lymphocytes, neutrophils, and macrophages
Glutamine	Regulates proliferation of lymphocytes, neutrophils, and macrophages Required for the production of various cytokines, such as IL-6, IFN-γ, and TNF-α

Abbreviations: Th, T helper cells; SCFAs, short-chain fatty acids; TLR, toll-like receptor; SARS, severe acute respiratory syndrome; IFN, interferon; TNF, tumor necrosis factor; IL, interleukin.

## Data Availability

Not Applicable.

## References

[1] Bourke C.D., Berkley J.A., Prendergast A.J. (2016). Immune Dysfunction as a Cause and Consequence of Malnutrition. Trends. Immunol..

[2] Katona P., Katona-Apte J. (2008). The interaction between nutrition and infection. Clin. Infect. Dis..

[3] Chapman I.M. (2006). Nutritional disorders in the elderly. Med. Clin. N. Am..

[4] Eggersdorfer M., Akobundu U., Bailey R.L., Shlisky J., Beaudreault A.R., Bergeron G., Blancato R.B., Blumberg J.B., Bourassa M.W., Gomes F. (2018). Hidden Hunger: Solutions for America’s Aging Populations. Nutrients.

[5] Samartin S., Chandra R. (2001). Obesity, overnutrition and the immune system. Nutr. Res..

[6] Childs C.E., Calder P.C., Miles E.A. (2019). Diet and Immune Function. Nutrients.

[7] Iddir M., Brito A., Dingeo G., Fernandez Del Campo S.S., Samouda H., La Frano M.R., Bohn T. (2020). Strengthening the Immune System and Reducing Inflammation and Oxidative Stress through Diet and Nutrition: Considerations during the COVID-19 Crisis. Nutrients.

[8] Ashby N.J.S. (2020). Impact of the COVID-19 Pandemic on Unhealthy Eating in Populations with Obesity. Obesity.

[9] Di Renzo L., Gualtieri P., Pivari F., Soldati L., Attinà A., Cinelli G., Leggeri C., Caparello G., Barrea L., Scerbo F. (2020). Eating habits and lifestyle changes during COVID-19 lockdown: An Italian survey. J. Transl. Med..

[10] Butler M.J., Barrientos R.M. (2020). The impact of nutrition on COVID-19 susceptibility and long-term consequences. Brain Behav. Immun..

[11] Delves P.J., Roitt I.M. (2000). The immune system. First of two parts. N. Engl. J. Med..

[12] Wintergerst E.S., Maggini S., Hornig D.H. (2007). Contribution of selected vitamins and trace elements to immune function. Ann. Nutr. Metab..

[13] Murphy K., Casey W. (2017). Janeway’s Immunobiology.

[14] Calder P.C., Kew S. (2002). The immune system: A target for functional foods?. Br. J. Nutr..

[15] Souza-Fonseca-Guimaraes F., Adib-Conquy M., Cavaillon J.M. (2012). Natural killer (NK) cells in antibacterial innate immunity: Angels or devils?. Mol. Med..

[16] Uribe-Querol E., Rosales C. (2020). Phagocytosis: Our Current Understanding of a Universal Biological Process. Front. Immunol..

[17] Romagnani S. (2000). T-cell subsets (Th1 versus Th2). Ann. Allergy Asthma Immunol..

[18] Zhu J., Yamane H., Paul W.E. (2010). Differentiation of effector CD4 T cell populations. Ann. Rev. Immunol..

[19] Spellberg B., Edwards J.E. (2001). Type 1/Type 2 immunity in infectious diseases. Clin. Infect. Dis..

[20] Libby P. (2007). Inflammatory mechanisms: The molecular basis of inflammation and disease. Nutr. Rev..

[21] Albers R., Antoine J.M., Bourdet-Sicard R., Calder P.C., Gleeson M., Lesourd B., Samartín S., Sanderson I.R., Van Loo J., Vas Dias F.W. (2005). Markers to measure immunomodulation in human nutrition intervention studies. Br. J. Nutr..

[22] Knight J.A. (2000). Review: Free radicals, antioxidants, and the immune system. Ann. Clin. Lab. Sci..

[23] Rodríguez L., Cervantes E., Ortiz R. (2011). Malnutrition and gastrointestinal and respiratory infections in children: A public health problem. Int. J. Environ. Res. Public Health.

[24] Andersen C.J., Murphy K.E., Fernandez M.L. (2016). Impact of Obesity and Metabolic Syndrome on Immunity. Adv. Nutr..

[25] Calder P.C., Carr A.C., Gombart A.F., Eggersdorfer M. (2020). Optimal Nutritional Status for a Well-Functioning Immune System Is an Important Factor to Protect against Viral Infections. Nutrients.

[26] Jantan I., Ahmad W., Bukhari S.N. (2015). Plant-derived immunomodulators: An insight on their preclinical evaluation and clinical trials. Front. Plant Sci..

[27] Gupta S., Brazier A.K.M., Lowe N.M. (2020). Zinc deficiency in low- and middle-income countries: Prevalence and approaches for mitigation. J. Hum Nutri. Diet..

[28] Huang Q., Wang L., Jiang H., Wang H., Zhang B., Zhang J., Jia X., Wang Z. (2020). Intra-Individual Double Burden of Malnutrition among Adults in China: Evidence from the China Health and Nutrition Survey 2015. Nutrients.

[29] Cowan A.E., Jun S., Tooze J.A., Eicher-Miller H.A., Dodd K.W., Gahche J.J., Guenther P.M., Dwyer J.T., Potischman N., Bhadra A. (2019). Total Usual Micronutrient Intakes Compared to the Dietary Reference Intakes among U.S. Adults by Food Security Status. Nutrients.

[30] Rippin H.L., Hutchinson J., Jewell J., Breda J.J., Cade J.E. (2017). Adult Nutrient Intakes from Current National Dietary Surveys of European Populations. Nutrients.

[31] Stewart D., McDougall G. (2014). Oat agriculture, cultivation and breeding targets: Implications for human nutrition and health. Br. J. Nutr..

[32] Alkhatib A. (2020). Antiviral Functional Foods and Exercise Lifestyle Prevention of Coronavirus. Nutrients.

[33] Pavadhgul P., Bumrungpert A., Harjani Y., Kurilich A. (2019). Oat porridge consumption alleviates markers of inflammation and oxidative stress in hypercholesterolemic adults. Asia Pac. J. Clin. Nutr..

[34] Gulvady A.A., Brown R.C., Bell J.A., Chu Y. (2013). Nutritional Comparison of Oats and Other Commonly Consumed Whole Grains. Oats Nutrition and Technology.

[35] Menon R., Gonzalez T., Ferruzzi M., Jackson E., Winderl D., Watson J., Henry J. (2016). Chapter One—Oats—From Farm to Fork. Advances in Food and Nutrition Research.

[36] Welch R.W., Webster F., Wood P.J. (2011). Nutrient Composition and Nutritional Quality of Oats and Comparisons with Other Cereals. Oats: Chemistry and Technology.

[37] Englyst H.N., Bingham S.A., Runswick S.A., Collinson E., Cummings J.H. (1989). Dietary fibre (non-starch polysaccharides) in cereal products. J. Hum. Nutr. Diet..

[38] Singh R., De S., Belkheir A. (2013). Avena sativa (Oat), a potential neutraceutical and therapeutic agent: An overview. Crit. Rev. Food Sci. Nutr..

[39] FDA, U Specific Requirements for Nutrient Content Claims. Code of Federal Regulation Title 21-FOOD AND DRUGS. https://www.accessdata.fda.gov/scripts/cdrh/cfdocs/cfcfr/CFRSearch.cfm?CFRPart=101&showFR=1&subpartNode=21:2.0.1.1.2.4.

[40] European Commission of the EU Nutrition Claims. https://ec.europa.eu/food/safety/labelling_nutrition/claims/nutrition_claims_en.

[41] Soycan G., Schär M.Y., Kristek A., Boberska J., Alsharif S.N.S., Corona G., Shewry P.R., Spencer J.P.E. (2019). Composition and content of phenolic acids and avenanthramides in commercial oat products: Are oats an important polyphenol source for consumers?. Food Chem..

[42] Bhavadharini B., Mohan V., Dehghan M., Rangarajan S., Swaminathan S., Rosengren A., Wielgosz A., Avezum A., Lopez-Jaramillo P., Lanas F. (2020). White Rice Intake and Incident Diabetes: A Study of 132,373 Participants in 21 Countries. Diabetes Care.

[43] Newby P.K., Muller D., Hallfrisch J., Qiao N., Andres R., Tucker K.L. (2003). Dietary patterns and changes in body mass index and waist circumference in adults. Am. J. Clin. Nutr..

[44] Jones J.M. (2013). Dietary Fiber Future Directions: Integrating New Definitions and Findings to Inform Nutrition Research and Communication. Adv. Nutr..

[45] Yang H., Sun Y., Cai R., Chen Y., Gu B. (2020). The impact of dietary fiber and probiotics in infectious diseases. Microb. Pathog..

[46] Shinde T., Hansbro P.M., Sohal S.S., Dingle P., Eri R., Stanley R. (2020). Microbiota Modulating Nutritional Approaches to Countering the Effects of Viral Respiratory Infections Including SARS-CoV-2 through Promoting Metabolic and Immune Fitness with Probiotics and Plant Bioactives. Microorganisms.

[47] Neuhouser M.L., Schwarz Y., Wang C., Breymeyer K., Coronado G., Wang C.-Y., Noar K., Song X., Lampe J.W. (2012). A low-glycemic load diet reduces serum C-reactive protein and modestly increases adiponectin in overweight and obese adults. J. Nutr..

[48] Liu H., Wang J., He T., Becker S., Zhang G., Li D., Ma X. (2018). Butyrate: A Double-Edged Sword for Health?. Adv. Nutr..

[49] Sun Y., O’Riordan M.X.D., Sariaslani S., Gadd G.M. (2013). Chapter Three—Regulation of Bacterial Pathogenesis by Intestinal Short-Chain Fatty Acids. Advances in Applied Microbiology.

[50] Park Y., Subar A.F., Hollenbeck A., Schatzkin A. (2011). Dietary fiber intake and mortality in the NIH-AARP diet and health study. Arch. Intern. Med..

[51] Budden K.F., Gellatly S.L., Wood D.L.A., Cooper M.A., Morrison M., Hugenholtz P., Hansbro P.M. (2017). Emerging pathogenic links between microbiota and the gut–lung axis. Nat. Rev. Microbiol..

[52] Jespersen L., Tarnow I., Eskesen D., Morberg C.M., Michelsen B., Bügel S., Dragsted L.O., Rijkers G.T., Calder P.C. (2015). Effect of Lactobacillus paracasei subsp. paracasei, L. casei 431 on immune response to influenza vaccination and upper respiratory tract infections in healthy adult volunteers: A randomized, double-blind, placebo-controlled, parallel-group study. Am. J. Clin. Nutr..

[53] King S., Glanville J., Sanders M.E., Fitzgerald A., Varley D. (2014). Effectiveness of probiotics on the duration of illness in healthy children and adults who develop common acute respiratory infectious conditions: A systematic review and meta-analysis. Br. J. Nutr..

[54] Yeh T.L., Shih P.C., Liu S.J., Lin C.H., Liu J.M., Lei W.T., Lin C.Y. (2018). The influence of prebiotic or probiotic supplementation on antibody titers after influenza vaccination: A systematic review and meta-analysis of randomized controlled trials. Drug Des. Dev. Ther..

[55] van den Munckhof I.C.L., Kurilshikov A., Ter Horst R., Riksen N.P., Joosten L.A.B., Zhernakova A., Fu J., Keating S.T., Netea M.G., de Graaf J. (2018). Role of gut microbiota in chronic low-grade inflammation as potential driver for atherosclerotic cardiovascular disease: A systematic review of human studies. Obes. Rev..

[56] Myhrstad M.C.W., Tunsjø H., Charnock C., Telle-Hansen V.H. (2020). Dietary Fiber, Gut Microbiota, and Metabolic Regulation-Current Status in Human Randomized Trials. Nutrients.

[57] Carlson J.L., Erickson J.M., Lloyd B.B., Slavin J.L. (2018). Health Effects and Sources of Prebiotic Dietary Fiber. Curr. Dev. Nutr..

[58] Villena J., Kitazawa H. (2020). The Modulation of Mucosal Antiviral Immunity by Immunobiotics: Could They Offer Any Benefit in the SARS-CoV-2 Pandemic?. Front. Physiol..

[59] Akramiene D., Kondrotas A., Didziapetriene J., Kevelaitis E. (2007). Effects of beta-glucans on the immune system. Medicina (Kaunas).

[60] Stier H., Ebbeskotte V., Gruenwald J. (2014). Immune-modulatory effects of dietary Yeast Beta-1,3/1,6-D-glucan. Nutr. J..

[61] Pan W., Hao S., Zheng M., Lin D., Jiang P., Zhao J., Shi H., Yang X., Li X., Yu Y. (2020). Oat-Derived β-Glucans Induced Trained Immunity Through Metabolic Reprogramming. Inflammation.

[62] Nieman D.C., Henson D.A., McMahon M., Wrieden J.L., Davis J.M., Murphy E.A., Gross S.J., McAnulty L.S., Dumke C.L. (2008). Beta-glucan, immune function, and upper respiratory tract infections in athletes. Med. Sci. Sports Exerc..

[63] Murphy E.A., Davis J.M., Brown A.S., Carmichael M.D., Carson J.A., Rooijen N.V., Ghaffar A., Mayer E.P. (2008). Benefits of oat β-glucan on respiratory infection following exercise stress: Role of lung macrophages. Am. J. Physiol. Regul. Integr. Comp. Physiol..

[64] Davis J.M., Murphy E.A., Brown A.S., Carmichael M.D., Ghaffar A., Mayer E.P. (2004). Effects of oat beta-glucan on innate immunity and infection after exercise stress. Med. Sci. Sports Exerc..

[65] Yun C.H., Estrada A., Van Kessel A., Park B.C., Laarveld B. (2003). Beta-glucan, extracted from oat, enhances disease resistance against bacterial and parasitic infections. FEMS Immunol. Med. Microbiol..

[66] Kristek A., Wiese M., Heuer P., Kosik O., Schär M.Y., Soycan G., Alsharif S., Kuhnle G.G.C., Walton G., Spencer J.P.E. (2019). Oat bran, but not its isolated bioactive β-glucans or polyphenols, have a bifidogenic effect in an in vitro fermentation model of the gut microbiota. Br. J. Nutr..

[67] Ganda Mall J.P., Fart F., Sabet J.A., Lindqvist C.M., Nestestog R., Hegge F.T., Keita Å.V., Brummer R.J., Schoultz I. (2020). Effects of Dietary Fibres on Acute Indomethacin-Induced Intestinal Hyperpermeability in the Elderly: A Randomised Placebo Controlled Parallel Clinical Trial. Nutrients.

[68] Queenan K.M., Stewart M.L., Smith K.N., Thomas W., Fulcher R.G., Slavin J.L. (2007). Concentrated oat beta-glucan, a fermentable fiber, lowers serum cholesterol in hypercholesterolemic adults in a randomized controlled trial. Nutr. J..

[69] Theuwissen E., Plat J., Mensink R.P. (2009). Consumption of oat beta-glucan with or without plant stanols did not influence inflammatory markers in hypercholesterolemic subjects. Mol. Nutr. Food. Res..

[70] Holscher H.D. (2017). Dietary fiber and prebiotics and the gastrointestinal microbiota. Gut Microbes.

[71] Di Caro V., Cummings J.L., Alcamo A.M., Piganelli J.D., Clark R.S.B., Morowitz M.J., Aneja R.K. (2019). Dietary Cellulose Supplementation Modulates the Immune Response in a Murine Endotoxemia Model. Shock.

[72] Honsek C., Kabisch S., Kemper M., Gerbracht C., Arafat A.M., Birkenfeld A.L., Dambeck U., Osterhoff M.A., Weickert M.O., Pfeiffer A.F.H. (2018). Fibre supplementation for the prevention of type 2 diabetes and improvement of glucose metabolism: The randomised controlled Optimal Fibre Trial (OptiFiT). Diabetologia.

[73] Kabisch S., Meyer N.M.T., Honsek C., Gerbracht C., Dambeck U., Kemper M., Osterhoff M.A., Birkenfeld A.L., Arafat A.M., Weickert M.O. (2019). Obesity Does Not Modulate the Glycometabolic Benefit of Insoluble Cereal Fibre in Subjects with Prediabetes-A Stratified Post Hoc Analysis of the Optimal Fibre Trial (OptiFiT). Nutrients.

[74] Stephen A.M., Champ M.M., Cloran S.J., Fleith M., van Lieshout L., Mejborn H., Burley V.J. (2017). Dietary fibre in Europe: Current state of knowledge on definitions, sources, recommendations, intakes and relationships to health. Nutr. Res. Rev..

[75] North C.J., Venter C.S., Jerling J.C. (2009). The effects of dietary fibre on C-reactive protein, an inflammation marker predicting cardiovascular disease. Eur. J. Clin. Nutr..

[76] EFSA Panel on Dietetic Products (2009). Scientific Opinion on the substantiation of health claims related to copper and protection of DNA, proteins and lipids from oxidative damage (ID 263, 1726), function of the immune system (ID 264), maintenance of connective tissues (ID 265, 271, 1722), energy yielding metabolism (ID 266), function of the nervous system (ID 267), maintenance of skin and hair pigment (ID 268, 1724), iron transport (ID 269, 270, 1727), cholesterol metabolism (ID 369), and glucose metabolism (ID 369) pursuant to Article 13(1) of Regulation (EC) No 1924/2006 on request from the European Commission. EFSA J..

[77] EFSA Panel on Dietetic Products (2009). Scientific Opinion on the substantiation of health claims related to zinc and function of the immune system (ID 291, 1757), DNA synthesis and cell division (ID 292, 1759), protection of DNA, proteins and lipids from oxidative damage (ID 294, 1758), maintenance of bone (ID 295, 1756), cognitive function (ID 296), fertility and reproduction (ID 297, 300), reproductive development (ID 298), muscle function (ID 299), metabolism of fatty acids (ID 302), maintenance of joints (ID 305), function of the heart and blood vessels (ID 306), prostate function (ID 307), thyroid function (ID 308), acid-base metabolism (ID 360), vitamin A metabolism (ID 361) and maintenance of vision (ID 361) pursuant to Article 13 of Regulation (EC) No 1924/2006 on request from European Commission. EFSA J..

[78] EFSA Panel on Dietetic Products (2016). Iron and contribution to the normal function of the immune system: Evaluation of a health claim pursuantto Article 14 of Regulation (EC) No 1924/2006. EFSA J..

[79] Caulfield L.E., Black R.E. (2004). Zinc deficiency. Comparative Quantification of Health Risks: Global and Regional Burden of Disease Attributable to Selected Major Risk Factors.

[80] Stoltzfus R.J. (2003). Iron deficiency: Global prevalence and consequences. Food Nutr. Bull..

[81] Micronutrients Dietary Reference Intakes for Vitamin A, Vitamin K, Arsenic, Boron, Chromium, Copper, Iodine, Iron, Manganese, Molybdenum, Nickel, Silicon, Vanadium, and Zinc. https://www.ncbi.nlm.nih.gov/books/NBK222312/.

[82] Awika J.M., Awika J.M., Piironen V., Bean S. (2011). Major Cereal Grains Production and Use around the World. Advances in Cereal Science: Implications to Food Processing and Health Promotion.

[83] Hurrell R.F., Reddy M.B., Juillerat M.A., Cook J.D. (2003). Degradation of phytic acid in cereal porridges improves iron absorption by human subjects. Am. J. Clin. Nutr..

[84] Tsopmo A., Preedy V. (2015). Chapter 43—Processing Oats and Bioactive Components. Processing and Impact on Active Components in Food.

[85] Larsson M., Rossander-Hulthén L., Sandström B., Sandberg A.S. (1996). Improved zinc and iron absorption from breakfast meals containing malted oats with reduced phytate content. Br. J. Nutr..

[86] Gupta R.K., Gangoliya S.S., Singh N.K. (2015). Reduction of phytic acid and enhancement of bioavailable micronutrients in food grains. J. Food Sci. Technol..

[87] Steinbrenner H., Al-Quraishy S., Dkhil M.A., Wunderlich F., Sies H. (2015). Dietary selenium in adjuvant therapy of viral and bacterial infections. Adv. Nutr..

[88] Im J.H., Je Y.S., Baek J., Chung M.H., Kwon H.Y., Lee J.S. (2020). Nutritional status of patients with coronavirus disease 2019 (COVID-19). Int. J. Infect. Dis..

[89] Avery J.C., Hoffmann P.R. (2018). Selenium, Selenoproteins, and Immunity. Nutrients.

[90] Beck M.A., Nelson H.K., Shi Q., Van Dael P., Schiffrin E.J., Blum S., Barclay D., Levander O.A. (2001). Selenium deficiency increases the pathology of an influenza virus infection. FASEB J..

[91] Combs G.F. (2001). Selenium in global food systems. Br. J. Nutr..

[92] (2002). Selenium. Human Vitamin and Mineral Requirements.

[93] White P.J. (2015). Selenium accumulation by plants. Ann. Bot..

[94] Yan L., Johnson L.K. (2011). Selenium bioavailability from naturally produced high-selenium peas and oats in selenium-deficient rats. J. Agric. Food Chem..

[95] Moreda-Piñeiro J., Moreda-Piñeiro A., Bermejo-Barrera P. (2017). In vivo and in vitro testing for selenium and selenium compounds bioavailability assessment in foodstuff. Crit. Rev. Food Sci. Nutr..

[96] Vanegas S.M., Meydani M., Barnett J.B., Goldin B., Kane A., Rasmussen H., Brown C., Vangay P., Knights D., Jonnalagadda S. (2017). Substituting whole grains for refined grains in a 6-wk randomized trial has a modest effect on gut microbiota and immune and inflammatory markers of healthy adults. Am. J. Clin. Nutr..

[97] Ampatzoglou A., Williams C.L., Atwal K.K., Maidens C.M., Ross A.B., Thielecke F., Jonnalagadda S.S., Kennedy O.B., Yaqoob P. (2016). Effects of increased wholegrain consumption on immune and inflammatory markers in healthy low habitual wholegrain consumers. Eur. J. Nutr..

[98] Leri M., Scuto M., Ontario M.L., Calabrese V., Calabrese E.J., Bucciantini M., Stefani M. (2020). Healthy Effects of Plant Polyphenols: Molecular Mechanisms. Int. J. Mol. Sci..

[99] Hachimura S., Totsuka M., Hosono A. (2018). Immunomodulation by food: Impact on gut immunity and immune cell function. Biosci. Biotechnol. Biochem..

[100] González-Gallego J., Sánchez-Campos S., Tuñón M.J. (2007). Anti-inflammatory properties of dietary flavonoids. Nutr. Hosp..

[101] Sahoo M., Jena L., Rath S.N., Kumar S. (2016). Identification of Suitable Natural Inhibitor against Influenza A (H1N1) Neuraminidase Protein by Molecular Docking. Genom. Inform..

[102] Kaul T.N., Middleton E., Ogra P.L. (1985). Antiviral effect of flavonoids on human viruses. J. Med. Virol..

[103] Roll S., Nocon M., Willich S.N. (2011). Reduction of common cold symptoms by encapsulated juice powder concentrate of fruits and vegetables: A randomised, double-blind, placebo-controlled trial. Br. J. Nutr..

[104] Droebner K., Ehrhardt C., Poetter A., Ludwig S., Planz O. (2007). CYSTUS052, a polyphenol-rich plant extract, exerts anti-influenza virus activity in mice. Antivir. Res..

[105] Harms L.M., Scalbert A., Zamora-Ros R., Rinaldi S., Jenab M., Murphy N., Achaintre D., Tjønneland A., Olsen A., Overvad K. (2020). Plasma polyphenols associated with lower high-sensitivity C-reactive protein concentrations: A cross-sectional study within the European Prospective Investigation into Cancer and Nutrition (EPIC) cohort. Br. J. Nutr..

[106] Zhu Y., Shao Y., Qu X., Guo J., Yang J., Zhou Z., Wang S. (2019). Sodium ferulate protects against influenza virus infection by activation of the TLR7/9-MyD88-IRF7 signaling pathway and inhibition of the NF-κB signaling pathway. Biochem. Biophys. Res. Commun..

[107] Zhang S., Wang P., Zhao P., Wang D., Zhang Y., Wang J., Chen L., Guo W., Gao H., Jiao Y. (2018). Pretreatment of ferulic acid attenuates inflammation and oxidative stress in a rat model of lipopolysaccharide-induced acute respiratory distress syndrome. Int. J. Immunopathol. Pharmacol..

[108] Wu Y.H., Zhang B.Y., Qiu L.P., Guan R.F., Ye Z.H., Yu X.P. (2017). Structure Properties and Mechanisms of Action of Naturally Originated Phenolic Acids and Their Derivatives against Human Viral Infections. Curr. Med. Chem..

[109] Bumrungpert A., Lilitchan S., Tuntipopipat S., Tirawanchai N., Komindr S. (2018). Ferulic Acid Supplementation Improves Lipid Profiles, Oxidative Stress, and Inflammatory Status in Hyperlipidemic Subjects: A Randomized, Double-Blind, Placebo-Controlled Clinical Trial. Nutrients.

[110] Zhang T., Zhao T., Zhang Y., Liu T., Gagnon G., Ebrahim J., Johnson J., Chu Y.F., Ji L.L. (2020). Avenanthramide supplementation reduces eccentric exercise-induced inflammation in young men and women. J. Int. Soc. Sports Nutr..

[111] Koenig R., Dickman J.R., Kang C., Zhang T., Chu Y.F., Ji L.L. (2014). Avenanthramide supplementation attenuates exercise-induced inflammation in postmenopausal women. Nutr. J..

[112] Ferreira A.O., Polonini H.C., Dijkers E.C.F. (2020). Postulated Adjuvant Therapeutic Strategies for COVID-19. J. Pers. Med..

[113] Ma Z.C., Hong Q., Wang Y.G., Liang Q.D., Tan H.L., Xiao C.R., Tang X.L., Shao S., Zhou S.S., Gao Y. (2011). Ferulic acid induces heme oxygenase-1 via activation of ERK and Nrf2. Drug Discov. Ther..

[114] McCarty M.F., DiNicolantonio J.J. (2020). Nutraceuticals have potential for boosting the type 1 interferon response to RNA viruses including influenza and coronavirus. Prog. Cardiovasc. Dis..

[115] Kawabata K., Yoshioka Y., Terao J. (2019). Role of Intestinal Microbiota in the Bioavailability and Physiological Functions of Dietary Polyphenols. Molecules (Basel).

[116] Cardona F., Andrés-Lacueva C., Tulipani S., Tinahones F.J., Queipo-Ortuño M.I. (2013). Benefits of polyphenols on gut microbiota and implications in human health. J. Nutr. Biochem..

[117] Kaulmann A., Bohn T. (2016). Bioactivity of Polyphenols: Preventive and Adjuvant Strategies toward Reducing Inflammatory Bowel Diseases-Promises, Perspectives, and Pitfalls. Oxid. Med. Cell. Longev..

[118] Man A.W.C., Zhou Y., Xia N., Li H. (2020). Involvement of Gut Microbiota, Microbial Metabolites and Interaction with Polyphenol in Host Immunometabolism. Nutrients.

[119] Chu Y., Fleige L. (2019). Increasing Bioavailability of Iron with Avenanthramide 2c. U.S. Patent.

[120] Calder P.C. (2006). Branched-chain amino acids and immunity. J. Nutr..

[121] Fülöp T., Wagner J.R., Khalil A., Weber J., Trottier L., Payette H. (1999). Relationship between the response to influenza vaccination and the nutritional status in institutionalized elderly subjects. J. Gerontol. Biol. Sci. Med. Sci..

[122] European Union EU Register of Nutrition and Health Claims Made on Foods. https://ec.europa.eu/food/safety/labelling_nutrition/claims/register/public/?event=register.home.

[123] Cruzat V., Macedo Rogero M., Noel Keane K., Curi R., Newsholme P. (2018). Glutamine: Metabolism and Immune Function, Supplementation and Clinical Translation. Nutrients.

[124] Name J.J., Rodrigues Vasconcelos A., Remondi Souza A.C., Favaro W. (2020). Vitamin D, Zinc and Glutamine: Synergistic Action with OncoTherad Immunomodulator in Interferon Signaling and COVID-19. SSRN.

[125] McRae M.P. (2017). Therapeutic benefits of glutamine: An umbrella review of meta-analyses. Biomed. Rep..

[126] Shah A.M., Wang Z., Ma J. (2020). Glutamine Metabolism and Its Role in Immunity, a Comprehensive Review. Animals.

[127] Herreman L., Nommensen P., Pennings B., Laus M.C. (2020). Comprehensive overview of the quality of plant- And animal-sourced proteins based on the digestible indispensable amino acid score. Food Sci. Nutr..

[128] Pomeranz Y., Youngs V.L., Robbins G.S. Protein Content and Amino Acid Composition of oat Species and Tissues. http://online.cerealsgrains.org/publications/cc/backissues/1973/Documents/Chem50_702.pdf.

